# A chromosome-scale reference genome for *Spironucleus salmonicida*

**DOI:** 10.1038/s41597-022-01703-w

**Published:** 2022-09-24

**Authors:** Feifei Xu, Alejandro Jiménez-González, Zeynep Kurt, Ásgeir Ástvaldsson, Jan O. Andersson, Staffan G. Svärd

**Affiliations:** 1 Department of Cell and Molecular Biology, BMC, Box 596, Uppsala University, SE-751 24 Uppsala, Sweden; 2 Present Address: Department of Medical Cell Biology, BMC, Box 571, Uppsala University, SE-751 23 Uppsala, Sweden; 3 Present Address: Department of Microbiology, National Veterinary Institute, SE-751 89 Uppsala, Sweden

**Keywords:** Parasite genomics, Comparative genomics, Genome

## Abstract

*Spironucleus salmonicida* is a diplomonad causing systemic infection in salmon. The first *S. salmonicida* genome assembly was published 2014 and has been a valuable reference genome in protist research. However, the genome assembly is fragmented without assignment of the sequences to chromosomes. In our previous *Giardia* genome study, we have shown how a fragmented genome assembly can be improved with long-read sequencing technology complemented with optical maps. Combining Pacbio long-read sequencing technology and optical maps, we are presenting here this new *S. salmonicida* genome assembly in nine near-complete chromosomes with only three internal gaps at long repeats. This new genome assembly is not only more complete sequence-wise but also more complete at annotation level, providing more details into gene families, gene organizations and chromosomal structure. This near-complete reference genome will aid comparative genomics at chromosomal level, and serve as a valuable resource for the diplomonad community and protist research.

## Abstract (summary)

**Table:** 

Measurement(s)	genomic_DNA • sequence_assembly • sequence feature annotation
Technology Type(s)	SMRT Sequencing • sequence assembly process • sequence annotation
Sample Characteristic - Organism	Spironucleus salmonicida

## Background & Summary

*Spironucleus salmonicida* (“the salmonid killer”) causes systemic infections in farmed Atlantic salmon, Chinook salmon and Arctic char^1,2^, thus poses a threat to sustainable aquaculture. Outbreaks of spironucleosis in farmed Atlantic salmon, *Salmo salar*, is a recurring problem and causes mass mortality and economical loss in for example Northern Norway. Salmon infected with *S. salmonicida* develops internal haemorrhaging, splenomegaly and granulomatous lesions in the liver and spleen, and drug treatment is not possible. This makes studies of the parasite important in order to develop alternative strategies to manage the parasite^3^.

*S. salmonicida* belongs to diplomonads, a group of unicellular protists with two diploid nuclei bearing different life styles. There are parasitic diplomonads like *S. salmonicida*, for example *Giardia* species which cause diarrhoea in various animals including humans^4^. All members of the *Giardia* genus are strictly intestinal parasites, while *S. salmonicida* is a well-adapting pathogen that can colonize different sites in the host^5^. It was shown in our previous study that *S. salmonicida* possesses an extended metabolic repertoire and more extensive gene regulation, probably making it more adapted to cope with environmental fluctuations^6^. There are also free-living diplomonads like *Trepomonas* sp. PC1, and comparative genomics have shown that the free-living life style most likely is a secondary adaptation and evolved from its parasitic ancestor^7,8^.

We recently published two *Giardia* genome assemblies, *Giardia intestinalis* WB^9^ and *Giardia muris*^10^, in near-complete chromosomes using Pacbio reads alone or in combination with optical maps. With similar sequencing and assembly strategy, we obtain a high-quality reference genome of *S. salmonicida* in near-complete chromosomes. Diplomonad genomes in near-complete chromosomes make it possible to study gene organization at the chromosomal level, and provide ground for studying chromosomal evolution.

## Methods

### DNA preparation and sequencing

*S. salmonicida* (ATCC 50377), previously known as *Spironucleus barkhanus*^2^, was isolated from a muscle abscess in Atlantic salmon grown in Vesterålen Sea in northern Norway. Cells were obtained from American Type Culture Collection (ATCC) in 2008 and grown axenically in slanted polypropylene tubes using a modified liver digest yeast extract (LYI) medium^11^ at 16 °C. Stocks of the *S. salmonicida* ATCC isolate were cryopreserved in liquid nitrogen. DNA extraction was performed in 2015, new batch of cells were taken up from the cryopreserved stock, cultured until confluent and DNA extracted directly to ensure minimal accumulation of mutations. Around 10^8^ cells were harvested for DNA extraction using a phenol-chloroform extraction. The DNA was then purified using the Qiagen Genomic Tip 100/G according to the manufacturer protocol. The purified DNA was quantified using a Qubit fluorometer and quality checked using a NanoDrop and agarose gel electrophoresis. DNA was then stored at −20 °C and 40 *μ*g of gDNA was sent directly for sequencing at the Uppsala Genome Center hosted by the Science for Life Laboratory (Uppsala University). A 10 kbp PacBio library was generated following the standard SMRT bell construction protocol according to the manufacturer recommended protocol. The library was sequenced on 6 SMRT cells of the Pacbio RS II instrument using the P6-C4 chemistry, which generated 267,495 reads in 2.6 billion bases (Table 1) with an N50 length of 14.6 kbp.

**Table 1:** Comparison of the old and the new *S. salmonicida* genome assemblies.

	Old	New
Sequencing instrument	454 FLX (Illumina GA IIx*)	PacBio RS II
# Reads	2,125,386 (18,886,541)	267,495
# Bases (Gbp)	0.7 (3.8)	2.6
Coverage	40X (280X)	161X
Assembler	Celera Assembler v6.0	HGAP3
Optical mapping	—	+
# Chromosomes	—	9
Genome size (Mbp)	12.9	14.7
# Contigs	452	45
# Scaffolds	233	42
# Gaps	232	7
Gap size (kbp)	61	284
G + C%	33.4	33.5
ASH%	0.15	0.09
# Genes	8,067	8,661
# Pseudogenes	21	194
# Partial genes	267	6
Mean gene length (aa)	373	384
Coding density %	72.1	69.5
Mean intergenic region (bp)	421	460
Number of introns	4	4
tRNAs	145	162
5S rRNAs	5	40

*This Illumina reads were also used for base correction in the new genome assembly.

### Genome assembly

The genome was assembled using the same method described in the *G. intestinalis* WB genome publication^9^. HGAP^12^ was used for *de novo* genome assembly, followed by consensus sequence calling with Quiver^12^. Both programs are part of SMRT Analysis (v2.3.0) pipeline^12^ from Pacbio. This yielded 61 contigs.

**Fig. 1:**
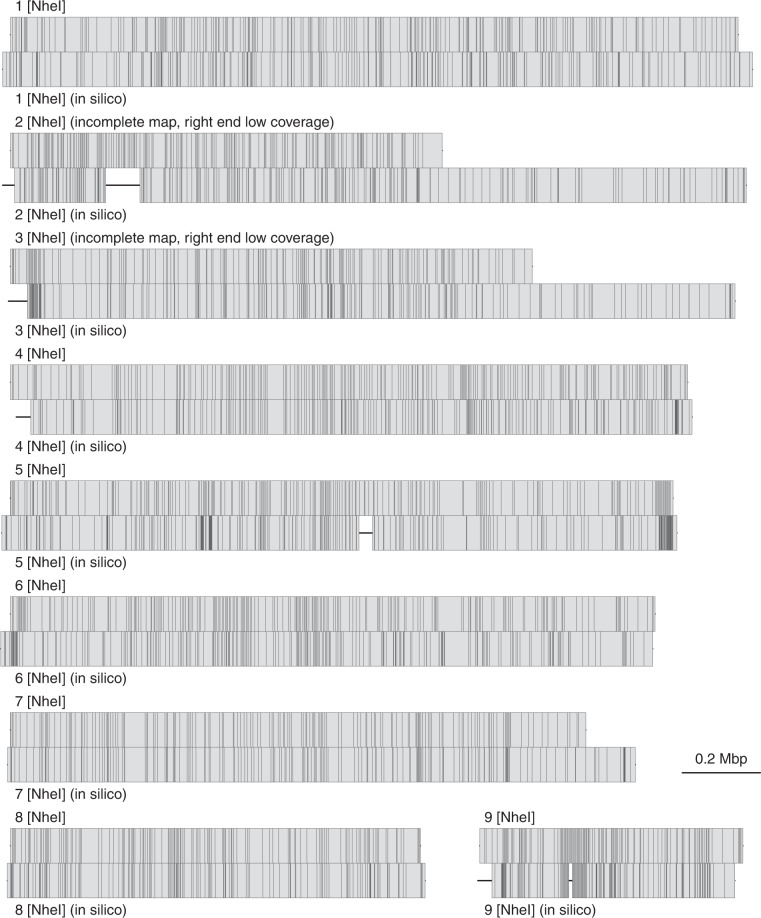
Nine near-complete chromosomes. Restriction enzyme (NheI) maps of the nine chromosomes aligned with the genomic sequences digested with NheI *in silico*. Each vertical line inside boxes represents a restriction enzyme cutting site. Gaps in the genomic sequences are represented with a horizontal line outside of boxes.

The contigs were then mapped to the optical maps of the nine chromosomes (Fig. 1, obtained for the old genome assembly in 2011^6^) using MapSolver (v3.2.0) from OpGen, and the mapping information was used to stitch together neighboring contigs into scaffolds, as described in the *G. intestinalis* WB genome publication^9^. To further close the gaps in the nine scaffolds, PBJelly (v15.8.24)^13^ was run using the Pacbio reads, and the resulting scaffolds were polished with Quiver^12^. Canu (v1.4)^14^ was used to assemble reads that failed to map to the scaffolds, which generated 33 contigs with sizes <  = 36 kbp. These small contigs do not map to the optical maps because they are below the size limitation for a sequence to uniquely map to an optical map. Another round of Quiver polishing was applied on Canu contigs combined with the nine scaffolds. To further improve per base quality, DNA Illumina paired-end reads (SRR948594^15^) were mapped to the draft assembly using BWA-MEM (v0.7.15)^16^ and the resulting BAM file was fed to Pilon (v1.21)^17^ to update indels and SNPs. The final assembly has a size of 14.7 Mbp, and is distributed in 42 scaffolds with nine major ones representing 96.5% of the total size (Table 1).

### Heterozygosity estimation

DNA Illumina reads (SRR948594^15^,) were re-mapped to the base-corrected sequences using BWA (v0.7.15)^16^ and samtools (v1.8)^18^ mpileup result was generated and parsed. Sites with at least 20X base coverage and an alternative base in at least 10% of the reads were called as SNP (or allelic sequence heterozygosity (ASH)) sites.

### Genome annotation

Annotation from the old *S. salmonicida* genome assembly^6^ was transferred to the new one using RATT (v0.95)^19^. 541 RATT transferred genes were selected to train GlimmerHMM (v3.0.1)^20^, which together with Prodigal (v2.6.3)^21^ were used for gene structural prediction. Functional annotation was performed using a combination of similarity information from BLASTP^22^ search against NR database and domain information from Conserved Domain (CD) search^23^. Transferred and predicted genes were then merged, and the inconsistent ones were manually inspected. RNA-seq reads (SRR948595^24^) mapped to the genome assembly were used as a guideline for structural annotation during manual examination, and the mapping was carried out with BWA (v0.7.15) since the old genome assembly contained only four introns. Searching for the conserved AC-repeat intron motif^6^ revealed no new intron in the new genome assembly.

The functional annotation was further improved by mining metabolic genes in Pathway Tools v21.5^25^ as described in *G. muris* genome publication^10^. An updated and curated *G. intestinalis* WB genome assembly^9^ has been published since the previous *S. salmonicida* genome was annotated. Genes shared with this assembly (BLASTP e-value <1*e*-10) but annotated differently were double checked to incorporate the updated annotations when applicable.

There are in total 8,661 protein-coding genes (plus an additional six partial and 194 pseudo genes) annotated in the new genome assembly (Table 1). Although the number of genes increased compared to the old genome assembly, with a bigger genome size and a bigger mean intergenic region size, coding density of the new genome turns out to be slightly smaller (Table 1).

**Fig. 2:**
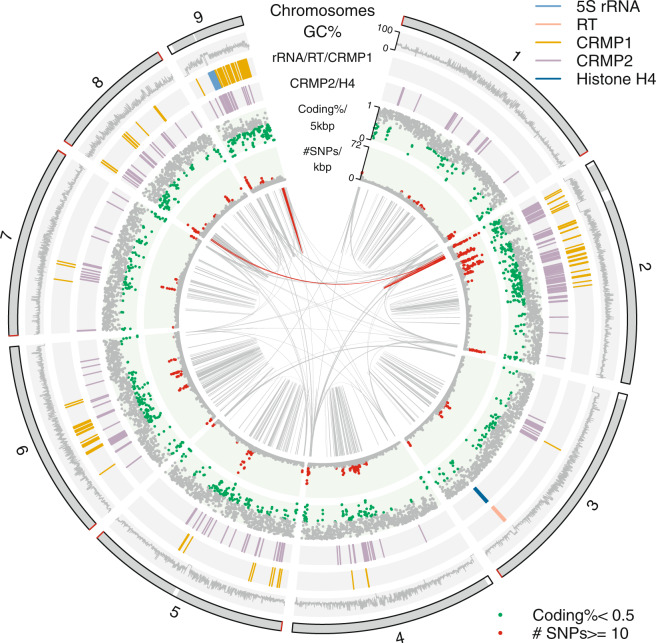
Circular plot of the nine chromosomes. Chromosomal sequences are represented in grey at the outermost circle with gaps in white bands and telomeres in red. Inner tracks are arranged as: GC%, 5 S rRNA/reverse transcriptase/CRMP1, CRMP2/Histone H4, coding density, SNPs density, regions with similarity. Regions with similarity represent BLASTN matches against itself with > = 95% sequence identity and > = 2000 bp in size, and two repetitive regions of 64 kbp in size are highlighted red. The circular plot was drawn with R package circlize (v0.4.8)^34^.

5 S ribosomal RNAs (rRNAs) were predicted using rnammer (v1.2)^26^, and there are 40 copies of them in tandem array located on chromosome 9 (Fig. 2). 18 S, 28 S and 5.8 S rRNA annotations were transferred from the old genome assembly, and there is one copy of each, all found in a single small contig just like in the old genome assembly^6^. In agreement with the old assembly, ribosomal RNA contig has a 15 times higher Pacbio read coverage compared to the genomic average, indicating the contig is most likely a collapse of repetitive reads. tRNAs were identified using tRNAscan-SE (v1.23)^27^, and there are more duplicated copies of tRNAs annotated in the new genome assembly (Table 1).

No sign of contamination has been observed at assembly nor annotation level.

## Data Records

This Whole Genome Shotgun project has been deposited at DDBJ/ENA/GenBank under the accession AUWU00000000. The version described in this paper is version AUWU02000000^28^, and the GenBank assembly accession is GCA_000497125.2^29^. Raw DNA sequence reads from Pacbio are deposited at NCBI Sequence Read Archive (SRA) under accession number SRP028565^30^. Pacbio reads mapped CRAM file (BAM file converted to CRAM file using samtools view command to reduce file size) is provided in the Figshare database under Digital Object Identifier (DOI) code^31^, and Illumina reads mapped BAM file is available in Figshare database under DOI code^32^.

## Technical Validation

### Genome completeness

**Fig. 3:**
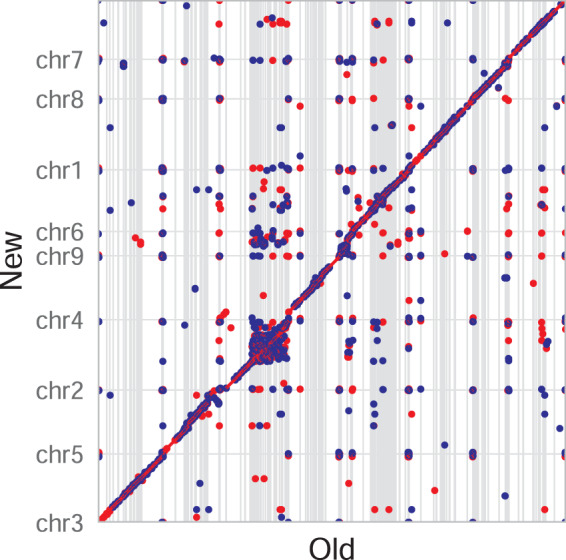
Dotplot of the nine chromosomes (new) vs. the old genome sequences. Blue represents forward matches while red represents reverse complement matches. MUMmer (v3.23)^35^ was used for the dotplot. DNA sequence alignment was generated using nucmer, and the alignment delta file was fed into mummerplot with ‘–layout’ turned on so that sequences are ordered and oriented in a way that the largest hits cluster near the main diagonal. Mplotter^36^ was then used to generate dots, lines and ticks from mummerplot output for drawing dotplot with R package ggplot2^37^.

Restriction enzyme (NheI) maps of the nine chromosomes align well with the genomic sequences digested with NheI *in silico* (Fig. 1). In fact, 95.2% of the optical maps are covered by the assembled genomic sequences. Interestingly, the right ends of chromosome 2 and 3 were reported as incomplete maps because of low coverage. However, long-read assembly extends well beyond the incomplete right ends of the optical maps, and the right end of chromosome 3 ends in telomeric repeats (TAGG)n. In fact, ten out of 18 chromosome ends are assembled into telomeric repeats, specially, chromosome 1, 5, 7 and 8 have both telomeric ends complete (Fig. 2). There are terminal gaps (Ns) at four out of the eight chromosome ends missing telomeric repeats, and the sizes were determined by the alignment to the optical maps. Three internal gaps also present in the new genome assembly, and the largest of all seven gaps is the internal gap located on chromosome 2 at a size of 87.9 kbp (Fig. 2). The new genome assembly is highly contiguous compared to the old fragmented genome assembly with 232 gaps distributed in 233 scaffolds, and preserves the synteny when compared to the old genome sequences (Fig. 3).

The new genome assembly is also better at resolving duplicated regions, which is defined as BLASTN matches against itself with >  = 2000 bp and 95% identity. In fact, 2.7 Mbp (19.2%) of the new genome sequences are characterized as duplicated regions compared to 1.5 Mbp of the old genome sequences. Regions involved in duplications are most commonly observed in short stretches of sequences with just one gene. However, we see also multi-gene regions involved in duplications up to 64 kbp in size (Fig. 2, ribbons highlighted in red in the innermost circle), which might be the largest repetitive regions the assembly could resolve due to the limitation of the read length. The largest BLASTN match is on chromosome 9, which is 33 kbp in size overlapping itself for 2 kbp resulting in a tandem repeat of 64 kbp in size (Fig. 2, red ribbon at chromosome 9 in the innermost circle). Manual examination of the Pacbio long read pileup and coverage at the two largest regions involved in duplications revealed the read coverage is in line with the neighbouring regions as well as the average coverage, indicating repetitive regions were correctly resolved. Genes found in duplicated regions are often members of multi-gene families. Duplicated regions also maintain higher GC contents with a mean GC level at 44.7%, while the rest of the genome sequence has a mean GC level at 30.7% and the whole genome sequence has a mean GC level at 33.5% (Table 1). Better resolved duplicated regions probably contribute to the observation of lower ASH level in the new genome assembly (Table 1).

### Annotation improvement

With this near-complete reference genome, we are able to present here a more complete annotation. Among the 8,661 full length protein-coding genes, 5,016 genes have exact copies as in the old genome assembly, 1,273 genes have the same sequences but updated descriptions, and 287 genes are the same but with SNPs. Start codons of 976 genes were adjusted, based on the alignments of orthologous genes and A-rich motifs at the start codons^6^. 3′ end sequences of 156 genes were updated due to differences of short insertions and deletions between the two genome sequences. 303 genes share sequence similarity with genes already annotated, but no distinct orthologous gene could be assigned. There are 650 genes which are unique to the new genome assembly, most of them are hypothetical proteins, but there are also genes with putative functional annotations. The most interesting unique gene is Histone H4, which was completely missing in the old genome assembly, but is presented in ten copies (plus five pseudo copies) in the new genome assembly with nine (plus the five pseudo copies) of them arranged in tandem array interspersed by four copies of reverse transcriptase (RT) on chromosome 3 (Fig. 2).

Better genome assembly leads also to better annotation of protein families. *S. salmonicida* harbors a large group of cysteine-rich membrane proteins, which were divided into cysteine-rich membrane protein 1 (CRMP1) and cysteine-rich membrane protein 2 (CRMP2)^6^. CRMP1s resemble variant-specific surface proteins (VSPs) in *G. intestinalis* structurally with a transmembrane domain at the 3′ end followed by a pentapeptide motif^6^, and VSPs are known to be expressed on the cell surface of the parasite to assist the parasite to bypass host immune system^33^. We find more CRMP1s and CRMP2s in the new genome assembly, now 138 CRMP1s (plus 22 pseudo and 1 partial) compared to 125 in the old genome assembly and 248 CRMP2s (plus 30 pseudo and 1 partial) compared to 195.

With nine near-complete chromosomes, we could visualize how these cysteine-rich membrane proteins are organized along the chromosomes (Fig. 2). The gene families are enriched in arrays on certain chromosomes, especially chromosome 2 (42 CRMP1s and 99 CRMP2s) and chromosome 9 (59 CRMP1s and 41 CRMP2s). This organization of gene families is different from what has been observed in the *Giardia* genomes. Cysteine-rich protein family specifically VSPs are enriched at terminal ends in *G. muris* genome^10^, while they are all over the chromosomes in *G. intestinalis* WB genome^9^. We also noticed that the regions around the cysteine-rich membrane proteins as well as the chromosomal ends tend to be gene-poor (Fig. 2, green dots in coding% track), and these regions tend to have higher allelic sequence variation (Fig. 2, red dots in SNPs track) and GC level. Similar patterns have been observed in the *G. intestinalis* WB genome^9^, suggesting that gene conversion mechanisms might be involved in the expansion of the antigenic surface protein repertoire.

## Data Availability

Software including their version information were listed in the method section. Custom scripts were provided at personal GitHub (https://github.com/feifei/scripts_to_share), including scripts to scaffold the contigs with optical maps (scaffolding_with_maps.py), to identify indel and SNPs using mapping information from Illumina DNA reads (base_change_from_pileup.py) and update reference sequence accordingly (base_change_incorporation.py), to call SNPs (snps.py), to analyze duplicated regions (duplicated_regions.py) and to generate the figures (optical_maps.R, circos.R, dotplot.R).
